# Socio-Economic Disparities in Attitude and Preference for Menu Labels among Vietnamese Restaurant Customers

**DOI:** 10.3390/ijerph15030460

**Published:** 2018-03-06

**Authors:** Long Hoang Nguyen, Bach Xuan Tran, Huong Lan Thi Nguyen, Huong Thi Le, Hoa Thi Do, Anh Kim Dang, Cuong Tat Nguyen, Carl A. Latkin, Melvyn W. B. Zhang, Roger C. M. Ho

**Affiliations:** 1 School of Medicine and Pharmacy, Vietnam National University, Hanoi 100000, Vietnam; longnh.ph@gmail.com; 2 Institute for Preventive Medicine and Public Health, Hanoi Medical University, Hanoi 100000, Vietnam; lethihuong@hmu.edu.vn (H.T.L.); dothihoa1954@yahoo.com (H.T.D.); kimanh.yhdp@gmail.com (A.K.D.); 3 Johns Hopkins Bloomberg School of Public Health, Baltimore, MD 21205, USA; Carl.Latkin@jhu.edu; 4 Vietnam Young Physicians’ Association, Hanoi 100000, Vietnam; 5 Institute for Global Health Innovations, Duy Tan University, Da Nang 550000, Vietnam; huong.ighi@gmail.com (H.L.T.N.); cuong.ighi@gmail.com (C.T.N.); 6 Biomedical Global Institute of Healthcare Research & Technology (BIGHEART), National University of Singapore, Singapore 117599, Singapore; melvynzhangweibin@gmail.com; 7 Department of Psychological Medicine, Yong Loo Lin School of Medicine, National University of Singapore, Singapore 119228, Singapore; hocmroger@yahoo.com.sg

**Keywords:** sociodemographic, disparity, calories, nutrients, labeling, Vietnam

## Abstract

Calories and nutrition labeling on restaurant menus are powerful policy interventions to reduce the burden of obesity epidemic. However, the success of this policy requires an assurance of equal benefits among customers with different characteristics, especially people at a higher risk of poor health outcomes and eating habits. This study examined the sociodemographic disparities in the attitude and preference for calories and nutrition labeling on menus among customers in various food facilities. A cross-sectional study was conducted with 1746 customers of food facilities in Hanoi, Vietnam, who were recruited by using a multistage sampling method. Socio-economic characteristics, attitudes regarding the necessity and preferences for calories, and nutrition labeling on menus were analyzed. Multivariate logistic regression was employed to determine the associated factors with attitudes and preferences. Results show that most of the sample understood the necessity to have calories and nutrition labeling (59.8%), and 71.8% preferred to have calories and nutrition labeling. People who often visited food facilities (Odd Ratio (OR) = 1.36; 95% confident interval (CI) = 1.06–1.74) and had higher education and were more likely to understand the necessity of calories and nutrition labeling. Factors such as being homemakers, often going to dine-in restaurants, and perceiving that labeling was unnecessary were negatively associated with preferences for calories and nutrition labeling. The results of this study encourage policymakers to implement calories and nutrition labeling in the future. Health education interventions to improve knowledge and attitude as regards calories and nutrition labeling on menus are important, particularly for males, less-educated individuals, and high-income people.

## 1. Introduction

Calories and nutrition labeling have been proposed as a cost-effective policy intervention against obesity and other malnutrition epidemics globally [1]. Informing nutrition contents (e.g., calories, nutrients, fat, etc.) to customers empowers them to purchase healthy food and have balanced diets [2,3]. This is especially important in settings where an increasing number of people are routinely eating in restaurants instead of at home. Food at these facilities has higher calories and poorer nutrients, and is often served in large portions, which may lead to overconsumption [4,5]. Previous studies indicated that calories and nutrition labeling on menus or menu boards at restaurants promoted customers’ food choices, increased their perceptions, and reduced their calories intake [6]. These positive effects could result in decreasing the burden of the obesity epidemic [7]. Thus, regulations requiring calories and nutrition labeling on menus have been implemented officially in some states of the U.S.A. and is of concern in other places such as the United Kingdom, China, and several Asian countries [8,9,10]. Global research has shown widespread interest from customers in seeing calories and nutrition labeling on menus or menu boards at restaurants, with about 50–70% of customers preferring to have and use calorie information on the menus [11,12,13].

Despite the rapid increase of obesity rates across all population groups, a remarkably higher burden was observed among people who were young, women, belonged to minority groups and had low income [14,15], leading to socio-economic disparities. These disparities are more likely to increase if public health policies cannot engage all demographic segments equally. Therefore, in order to become an effective public health tool, calories and nutrition labeling policies should assure equal benefits for consumers with different sociodemographic characteristics, particularly people who are at a higher risk of poor health and eating habits. Some prior studies found that female clients were more likely to use calorie information on the menus, while there were some mixed results according to age, education, and income groups [16,17,18,19].

The prevalence of overweight and obesity in Vietnam has been rising proportionately with the economic growth, especially in urban areas. Two national surveys indicated that the rate of individuals with overweight and obesity approximately doubled from 3.7% in 2000 to approximately 7% in 2005 [20]. A study in Ho Chi Minh city—a Vietnamese metropolis—in 2015 found that 24% males and 19% females were overweight and obesity [20]. Importantly, the occurrence of overweight and obesity is increasingly observed in Vietnamese preschool children and adolescents, which may be due to the expansion of fast-food restaurants, sedentary lifestyles, as well as the academic burden [21,22]. In 2010, the Vietnam National Assembly enacted the Law on Food Safety following the CodeX Alimentarius (a joint United Nations and World Health Organization Commission) guideline, requiring nutrition labeling on the pack of food products [8,23]. However, the law does not provide regulations for calories and nutrition labeling on the menus of food facilities [23]. Therefore, it is hard to find nutrition labels on the menus of Vietnamese restaurants.

Given the dearth of information about calories and nutrition labeling on the menus in Vietnam, this study examined the sociodemographic disparities in the attitudes and preferences regarding calories and nutrition labeling on the menus among customers in various food facilities. The result will be expected to partly contribute to developing nutritional strategies for alleviating the overweight and obesity epidemic in Vietnam.

## 2. Materials and Methods

### 2.1. Study Design

Participants were 1746 customers in fast-food restaurants, dine-in restaurants, street food restaurants, and other food facilities (such as cafeterias, street food vendors, etc.). They were recruited for a cross-sectional survey which was conducted in Hanoi from October to November 2015. Hanoi is the capital of Vietnam, having 577 communes clustered within 30 districts. According to the General Statistics Office in 2016, the population in Hanoi was young given that 52.2% of people were 15 years old or above. Most of the residents were female (51.0%) and living in urban areas (53.6%) [24]. In this study, the eligible criteria included: (1) aged ≥15 years old; (2) using food services in selected food facilities; and (3) provided informed consent to participate in this study.

We performed a multistage sampling method to recruit respondents. First, among 29 districts of Hanoi, we randomly selected 176 communes. Then, in each commune, we listed all food facilities that were registered with local authorities, and randomly picked ten facilities. Finally, the data collectors visited these facilities and recruited the third customer after them. A total of 1760 clients were invited to participate in the study, and data of 1746 customers were used for analysis (99.2%). We excluded data from 14 clients because they decided to withdraw during the interview.

### 2.2. Measures and Instruments

We constructed a structured questionnaire and piloted it with 20 consumers to validate the tools. After revision, the questionnaire was used by the data collection teams who were Master of Public Health students at Hanoi Medical University. These students were trained to collect the data consistently and ensure the quality of data. Respondents were interviewed face-to-face within 15–20 min.

The questionnaire included socioeconomic characteristics (age, gender, education attainment, marital status, living location, employment, monthly household income); self-reported height and weight; attitudes and preferences for calories and nutrition labeling on the menus in the restaurants.

Body mass index (BMI) was calculated by using height and weight data. People were classified into three groups according to the Asian standards [20]: underweight (<18.5 kg/m^2^); normal (18.5–24.9 kg/m^2^); and overweight/obesity (≥25 kg/m^2^).

For the attitudes and preferences, we asked respondents to report whether they frequently visited food facilities for food services, preferable types of food facilities (fast-food, dine-in restaurant, street food, or others), criteria for ordering food (name, nutrition, introduction, price or others), attitudes regarding the necessity of calories and nutrition labeling (with five-point Likert scale from ‘very unnecessary’ to ‘very necessary’). People were categorized into the ‘necessary’ group if they selected ‘very necessary’ or ‘necessary’; otherwise, they were belonged to ‘not necessary’ group. We also asked them about the preferences for having calories and nutrition labeling (‘yes/no’).

### 2.3. Statistical Analysis

We analyzed the data using STATA software version 12.0 (StataCorp. LP, College Station, TX, USA). *p*-value < 0.05 was used for identifying the statistical significance. We used a multivariate logistic regression to identify the associated factors with “Attitudes regarding the necessity of calories and nutrition labeling” (necessary/not necessary) and “Prefer to have calories and nutrition labeling” (yes/no). These models were combined with a forward stepwise selection strategy to produce the reduced models.

### 2.4. Ethics Approval

The study protocol was reviewed and approved by the IRB of the Hanoi Health Department (code: 06/CCATVSTPHN). We obtained the written informed consents from participants. Their data were only used for research and kept confidentially.

## 3. Results

Among 1746 participants, most of them were female (61.9%), aged from 26 to 39 years (41.4%), living with spouse/partners (64.3%), had higher education (56.0%), and were white-collar officers (30.6%). There were 18.6% respondents with obesity (Table 1).

Table 2 presents that 68.6% clients reported that they frequently visited food facilities. Street food restaurants were the preferable facility of 43.9% customers, following by the dine-in restaurants (42.2%) and fast food restaurants (41.2%). Name and nutrition of food were the two favorable criteria when ordering food (with 48.6% and 47.6%, respectively), followed by the introductory statement and price of food (with 43.1% and 21.5%, correspondingly). Most of the sample felt that it was necessary or very necessary to label nutrition on the menus (59.8%), and 71.8% preferred to have food label on the menus.

Table 3 shows that most of female customers perceived that menu labeling was necessary (63.8%) and preferred menu labels (74.9%). These rates were significantly higher than those in males (53.4% and 66.7%, respectively). People belonged to the age group ≥60 years (48.7%), being separated/divorced/widowed (40.0%), attaining < high school education (40.2%), and being blue-collar workers (45.9%) had the lowest percentages compared to other groups in having positive attitudes regarding menu labels. These tendencies were also observed in preferring to have menu labels. These differences were statistically significant (*p* < 0.05). Meanwhile, we did not find any statistically significant differences among income groups, living locations, BMI categories, and often food facilities visit (*p* > 0.05).

Table 4 shows that people who were male (OR = 0.54; 95% CI = 0.43–0.68), and had higher income were less likely to perceive that calories and nutrition labeling was necessary. Otherwise, often visiting food facility (OR = 1.38; 95% CI = 1.08–1.77) and having higher education had positive associations with feeling the necessity of calories and nutrition labeling. In addition, people who were homemakers/others, who often going to dine-in restaurants (OR = 0.38; 95% CI = 0.18–0.80) were less likely to prefer to have calories and nutrition labeling. Meanwhile, having positive attitudes with menu labeling was significantly associated with preferring to have menu labels (OR = 32.62; 95% CI = 21.96–48.46). Body mass index or overweight/obesity was not associated with attitudes and preferences for menu labels.

## 4. Discussion

The current study highlighted the positive attitudes and high demand for calories and nutrition labeling by consumers in food facilities in a Vietnamese urban setting. We also explored the existing socio-demographic disparities in the attitudes and preferences for calories and nutrition labeling in food facilities, which can potentially be used to develop interventions tailored for different groups of customers in the future.

In this study, we found that a high proportion of respondents understood the necessity of calories and nutrition labeling and preferred to have calories and nutrition labeling in the food facilities. These results were consistent with other findings, which demonstrated that calories and nutrition labeling had a widespread support from the public [11,12,13,25]. Importantly, the nutrition of food was the second most important information that the clients used for ordering food, and people who often visited food facilities were more likely to perceive the necessity for menu labels. Indeed, we found two-thirds of customers visited food facilities frequently, which might put them at higher risk of obesity. The literature suggested that people who have meals outside the home more than five times per week were more likely to be obese [26]. These results were very critical that our sample were aware of the importance of menu labeling intervention in protecting their health and preventing overweight/obesity. Therefore, adopting the low-cost tool such as posting calories information on menus should be considered to inform people about healthy food choice.

Our analysis indicated that there were disparities in the attitudes and preferences for calories and nutrition labeling in certain socio-demographic characteristics such as gender and education. Men were less likely to perceive the necessity of calories and nutrition labeling, which was similar to other studies [19,25,11]. Otherwise, women preferred restaurants having caloric information on the menus because this information could help them to choose the lower-calorie dishes and control their diets [25,11]. People who were well-educated were also observed to have a favorable response to calories and nutrition labeling in food facilities. This may be explained by the fact that people with higher education had a higher likelihood to have healthy behaviors (doing physical exercise, eating a healthy diet, not smoking or drinking alcohol, etc.) or seek health information frequently to have better health outcomes [27].

Nonetheless, although income was not associated with the attitudes regarding menu labeling in the univariate analysis, in the multivariate model, income was negatively related to the attitudes. This finding was different from previous studies, which found that wealthier people had more interested in calories and nutrition labeling [19]. The reason for this phenomenon was not clear. In fact, we observed that lower-income individuals were more likely to choose fast-food restaurants, while higher-income people were more likely to visit dine-in restaurants. In addition, people often selecting dine-in restaurants—albeit not statistically significant and not included in the final model—were less likely to prefer calories and nutrition labeling. We supposed that they believed the food in dine-in restaurants had more balanced and healthier nutrition compared to fast-food or street food restaurants, which made them feel that calories and nutrition labeling was not necessary [28].

The study findings suggest several implications. First, policymakers should consider implementing interventions requiring calories and nutrition labeling not only in fast-food restaurants but also in dine-in restaurants and other food facilities. Second, educational interventions about the importance of a healthy diet and the necessity of calories and nutrition labeling should be provided, particularly for male and high-income individuals, in order to encourage them to control their calories and nutrient intake. This, in turn, will help to control the obesity epidemic that is increasing in Vietnam. Finally, a study to examine the barriers and facilitators of implementing calories and nutrition labeling from the providers’ perspective should be conducted to provide a comprehensive view of the feasibility of this intervention.

This study has strengths in a large sample size with various types of restaurants selected. Nonetheless, several limitations should be pointed out. First, this study was conducted only in Hanoi, a metropolitan area of Vietnam. Moreover, the socio-demographic characteristics of respondents in this study were slightly different compared to these characteristics of the general population in Hanoi. Therefore, the result had a limited generalizability that might not apply to other settings. Second, the cross-sectional design does not allow us to identify the causal relations between attitudes and preferences and its associated factors. Finally, there are some features that we did not take into account in this study, such as the effective approach to communicate caloric and nutritional information. In addition, we could not test the effect of calories and nutrition labeling on the reduction of energy and nutrients consumed. Further studies should be conducted to fill these gaps.

## 5. Conclusions

In conclusion, the positive attitudes and preferences for calories and nutrition labeling by customers found in this study should inform actions to implement this intervention in the future. Educational interventions to improve knowledge and attitudes regarding calories and nutrition labeling are important, particularly for male, less educated individuals, and high-income people. Further research is needed to examine the opinions of food sellers and the most effective way for calories and nutrition labeling.

## Figures and Tables

**Table 1:** Demographic characteristics of customers and food sellers.

Characteristics	*n*	%
**Gender**		
Male	634	38.1
Female	1042	61.9
**Age group**		
<18 years	28	1.6
18–25 years	393	22.6
26–39 years	721	41.4
40–59 years	483	27.8
≥60 years	115	6.6
**Marital status**		
Single	591	34.0
Living with spouse/partner	1116	64.3
Separate/divorced/widowed	30	1.7
**Education**		
<High school	224	13.0
High school	535	31.1
>High school	964	56.0
**Occupations**		
Students	309	17.8
Blue-collar workers	304	17.5
White-collar officers	531	30.6
Homemakers	233	13.4
Others	361	20.8
**Living location**		
Urban	1443	82.9
Rural	297	17.1
**Categories of body mass index**		
Normal	1301	77.9
Underweight	60	3.6
Overweight/obesity	310	18.6
	**Mean**	**SD**
Monthly household income (million VND)	5.2	5.7

**Table 2:** Attitude and preference for calories and nutrition labeling among customers.

Characteristics	*n*	%
**Often visit food facilities**		
Yes	1178	68.6
No	539	31.4
**Regular choice of food facilities**		
Fast food restaurants	712	41.2
Dine-in restaurants	729	42.2
Street food restaurants	762	43.9
Others	135	8.1
**Selection criteria when ordering food at food facilities**		
Name of food	796	48.6
Nutrition of food	797	47.6
Introductory statement of food	360	21.5
Price of food	739	43.1
Others	149	8.9
**Attitude regarding the necessity of menu labels**		
Very necessary	234	13.9
Necessary	773	45.9
Neutral	415	24.6
Unnecessary	250	14.9
Very unnecessary	12	0.7
**Prefer to have menu labels**		
Yes	1213	71.8
No	477	28.2

**Table 3:** Socio-economic characteristics of respondents regarding attitudes and preferences for calories and nutrition labeling.

Characteristics	Attitude Regarding the Necessity of Menu Labels				*p*-Value	Prefer to Have Menu Labels				*p*-Value
	Not Necessary		Necessary			No		Yes		
	*n*	%	*n*	%		*n*	%	*n*	%	
**Gender**										
Female	377	36.3	663	63.7	<0.01	262	25.1	782	74.9	<0.01
Male	300	46.6	344	53.4		215	33.3	431	66.7	
**Age group**										
<18 years	10	35.7	18	64.3	<0.01	4	14.3	24	85.7	0.03
18–25 years	129	34.3	247	65.7		89	23.7	287	76.3	
26–39 years	274	39.1	427	60.9		200	28.4	505	71.6	
40–59 years	208	43.9	266	56.1		144	30.4	329	69.6	
≥60 years	57	51.4	54	48.6		41	36.0	73	64.0	
**Marital status**										
Single	207	36.5	360	63.5	0.01	141	24.8	427	75.2	<0.01
Living with spouse/partner	451	41.4	639	58.6		319	29.2	775	70.8	
Separate/divorced/widowed	18	60.0	12	40.0		16	53.3	14	46.7	
**Education**										
<High school	128	59.8	86	40.2	<0.01	98	45.2	119	54.8	<0.01
High school	224	42.8	299	57.2		153	29.1	372	70.9	
>High school	325	34.9	606	65.1		218	23.4	714	76.6	
**Occupations**										
Students	90	30.5	205	69.5	<0.01	59	19.9	237	80.1	<0.01
Blue-collar workers	159	54.1	135	45.9		107	36.0	190	64.0	
White-collar officers	185	35.6	335	64.4		114	21.9	407	78.1	
Homemakers	92	40.7	134	59.3		75	33.2	151	66.8	
Others	151	42.9	201	57.1		122	34.6	231	65.4	
**Income quintiles**										
Poorest	115	35.8	206	64.2	0.43	80	24.8	242	75.2	0.13
Poor	116	41.4	164	58.6		85	30.4	195	69.6	
Middle	200	40.7	292	59.3		132	26.7	362	73.3	
Rich	66	42.6	89	57.4		42	27.1	113	72.9	
Richest	102	42.9	136	57.1		81	34.0	157	66.0	
**Living location**										
Urban	100	39.2	155	60.8	0.75	73	28.4	184	71.6	0.93
Rural	578	40.3	857	59.7		405	28.1	1034	71.9	
**Body mass index**										
Normal	508	40.3	752	59.7	0.11	337	26.6	928	73.4	0.07
Underweight	23	39.0	36	61.0		20	33.9	39	66.1	
Overweight/obesity	142	46.9	161	53.1		99	32.6	205	67.4	
**Often visit food facilities**										
Yes	449	39.2	696	60.8	0.11	307	26.7	842	73.3	0.08
No	225	43.4	294	56.7		161	30.9	360	69.1	
**Regular choice of food facilities**										
Fast food restaurants	264	38.2	427	61.8	0.17	185	26.8	506	73.2	0.28
Dine-in restaurants	267	37.7	442	62.3	0.07	199	28.0	513	72.1	0.84
Street food restaurants	330	43.9	421	56.1	<0.01	231	30.6	523	69.4	0.04
Others	26	21.3	96	78.7	<0.01	31	25.4	911	74.6	0.58
**Attitude regarding the necessity of menu labels**										
Very necessary						7	2.9	231	97.1	<0.01
Necessary						55	7.1	718	92.9	
Neutral						170	41.1	244	58.9	
Unnecessary						232	92.8	18	7.2	
Very unnecessary						12	100.0	0	0.0	

**Table 4:** Associated factors with the attitude and preference for calories and nutrition labeling among customers

Factors	Attitude Regarding the Necessity of Menu Labels (Necessary/Not Necessary)		Prefer to Have Menu Labels (Yes/No)	
	OR	95% CI	OR	95% CI
**Gender (male vs. female)**	0.54 ***	0.43; 0.68	0.72	0.51; 1.01
**Marital status (vs. single)**				
**Live with spouse/partner**			1.22	0.80; 1.86
Divorced/widowed			0.37	0.10; 1.35
**Education attainment (vs. <high school)**				
High school	1.82 ***	1.26; 2.65		
>High school	2.64 ***	1.84; 3.79		
**Occupation (vs. students)**				
Blue-collar workers			0.56	0.30; 1.08
White-collar workers			0.76	0.42; 1.37
Homemakers			0.38 **	0.18; 0.80
Others			0.38 ***	0.20; 0.73
**Income quintiles (vs. poorest)**				
Poor	0.77	0.54; 1.10		
Middle	0.70 **	0.51; 0.96		
Rich	0.57 **	0.37; 0.88		
Richest	0.63 **	0.43; 0.93		
**Living location (urban vs. rural)**	1.27	0.91; 1.76		
**BMI categories (vs. normal)**				
Underweight			0.43	0.18; 1.00
Overweight/obesity			0.99	0.67; 1.46
**Often visit food facility (Yes vs. no)**	1.38 **	1.08; 1.77		
**Regular choice of food facilities (vs. no)**				
Fast food restaurants	0.85	0.67; 1.08		
Street food restaurants	0.83	0.66; 1.05		
**Attitude toward the necessity of food labeling** **(vs. not necessary)**				
Necessary			32.62 ***	21.96; 48.46
Pseudo R2	0.043		0.360	
Hosmer-Lemeshow chi2	6.32		3.61	
Prob > chi2	0.61		0.89	

*** *p* < 0.01, ** *p* < 0.05; OR: Odds ratio; CI: Confident interval.
